# Zinc biofortification of hydroponically grown basil: Stress physiological responses and impact on antioxidant secondary metabolites of genotypic variants

**DOI:** 10.3389/fpls.2022.1049004

**Published:** 2022-10-27

**Authors:** Michele Ciriello, Luigi Formisano, Marios Kyriacou, Georgios A. Soteriou, Giulia Graziani, Stefania De Pascale, Youssef Rouphael

**Affiliations:** ^1^ Department of Agricultural Sciences, University of Naples Federico II, Portici, Italy; ^2^ Department of Vegetable Crops, Agricultural Research Institute, Nicosia, Cyprus; ^3^ Department of Pharmacy, Faculty of Pharmacy, University of Naples “Federico II”, Naples, Italy

**Keywords:** *Ocimum basilicum* L., floating system, Zn agronomic biofortification, pigments, UHPLC, phenolics

## Abstract

*Ocimum basilicum* L. is an aromatic plant rich in bioactive metabolites beneficial to human health. The agronomic biofortification of basil with Zn could provide a practical and sustainable solution to address Zn deficiency in humans. Our research appraised the effects of biofortification implemented through nutrient solutions of different Zn concentration (12.5, 25.0, 37.5, and 50 µM) on the yield, physiological indices (net CO_2_ assimilation rate, transpiration, stomatal conductance, and chlorophyll fluorescence), quality, and Zn concentration of basil cultivars ‘Aroma 2’ and ‘Eleonora’ grown in a floating raft system. The ABTS, DPPH, and FRAP antioxidant activities were determined by UV-VIS spectrophotometry, the concentrations of phenolic acids by mass spectrometry using a Q Extractive Orbitrap LC-MS/MS, and tissue Zn concentration by inductively coupled plasma mass spectrometry. Although increasing the concentration of Zn in the nutrient solution significantly reduced the yield, this reduction was less evident in ‘Aroma 2’. However, regardless of cultivar, the use of the maximum dose of Zn (50 µM) increased the concentration of carotenoids, polyphenols, and antioxidant activity on average by 19.76, 14.57, and 33.72%, respectively, compared to the Control. The significant positive correlation between Zn in the nutrient solution and Zn in plant tissues underscores the suitability of basil for soilless biofortification programs.

## Introduction

A diversified and well-balanced diet based on high nutritional quality foods is prerequisite to good health and, according to the World Health Organization (WHO), it is dependent on the development of sustainable agricultural systems (Praharaj et al., 2021; Szerement et al., 2021). Insidious and invisible, the “hidden hunger” associated with micronutrient malnutrition (Fe, Zn, I, and Se) affects more than two billion people in underdeveloped areas even in the most industrialized countries (Buturi et al., 2021; Praharaj et al., 2021). The impact is so dramatic that, according to the World Bank, the economic cost of dealing with the problem is estimated to about 5% of a country’s gross domestic product (Sharma T.R. et al., 2020). Although the role of Zn in human nutrition has been known since 1961, its deficiency is widespread (Praharaj et al., 2021). Given its vital function in critical phases of growth, development, and reproduction, inadequate Zn intake jeopardizes the mental and physical well-being of adults and children by altering the immune, nervous, visual, gastrointestinal, and skeletal systems and increasing the incidence of infections and cancer (Hotz and Brown, 2004; Cakmak, 2008; Gibson, 2012; Roohani et al., 2013; Cakmak and Kutman, 2018). Although Zn deficiency is associated with overconsumption of processed foods and grains high in phytates, it should be noted that agricultural soils often limit bioaccumulation of this valuable mineral in agricultural products due to low phyto-availability or total deficiency (White and Broadley, 2011; Meneghelli et al., 2021). The link between agriculture and nutrition highlights how the mineral enrichment process of agricultural products, known as biofortification, is a practical and sustainable solution to Zn deficiency in humans, since most of the human diet is plant-based (Padash et al., 2016; Sharma T.R. et al., 2020). Through agronomic practice, genetic improvement, and genetic engineering strategies, biofortification can increase the bioavailability of essential trace elements in the edible parts of plants (Barrameda-Medina et al., 2017; Sharma T.R. et al., 2020). The agronomic approach, based on crop management and fertilization practices to improve the mobilization and uptake of microelements by plants, has been recognized as the most practical and user-friendly biofortification strategy (Buturi et al., 2021; Szerement et al., 2021). Agronomic biofortification of staple crops is often ineffective in providing adequate Zn intake due to the presence of antinutritional compounds (e.g., tannins and phytates) that suppress intestinal assimilation. In this regard, greater interest should be given to the biofortification of leafy vegetables because it facilitates higher Zn concentrations transported mainly through the xylem (Sahin, 2021). However, the results achievable by ordinary agronomic soil biofortification programs, either through fertilization or by foliar application, are severely influenced by interactions between genotype, environment, and soil characteristics, as well as nutrient interactions during uptake (Szerement et al., 2021).

From this point of view, the limitations of agronomic biofortification in soil cultivation can be overcome by using hydroponic growing systems where nutrient solutions with *ad hoc* Zn concentrations would allow a standardized, fine control of leafy vegetable quality as already observed in *Lactuca sativa* L., *Thlaspi caerulescens*, and *Brassica oleracea* (Zhao et al., 1998; Barrameda-Medina et al., 2014; Meneghelli et al., 2021). Furthermore, soilless growing systems could ensure efficient high-yield and high-quality production even under land-limiting (such as growing in urban areas) or prohibitive (contaminated soils and scarce water resources) environments (Raviv et al., 2008; Orsini et al., 2013; Bonasia et al., 2017; Buturi et al., 2021). A successful hydroponic biofortification program could also be implemented on aromatic herbs such as basil, to increase the concentration of desirable secondary metabolites (such as phenolic acids and volatile aroma compounds) that characterize the flavor of tender green leaves and constitute traits of premium quality that consistently attract the interest of producers and consumers (Ciriello et al., 2021b). Zn is also essential for plants to perform crucial metabolic functions. This micronutrient is an integral component of enzymes, involved in the synthesis and degradation of sugars, lipids, and nucleic acids, it regulates the translation and transcription of DNA, stabilizes proteins, repairs photosystems, and regulates the function of chloroplasts, oxidoreductases, and hydrolytic enzymes (White and Broadley, 2011; Padash et al., 2016; Sharma T.R. et al., 2020; Buturi et al., 2021). Roots take Zn primarily as Zn^2+^ through ZIP transporters or chelated with low molecular weight compounds (phytosiderophores), a mechanism typical only of *Poaceae* (Broadley et al., 2007; Sharma T.R. et al., 2020). In the plant, Zn is carried through the xylem either symplastically or apoplastically in its ionic form or bound with organic acids, histidine or nicotianamine (White and Broadley, 2009) and the differences in concentration in the edible parts may depend both on the mode of uptake and on the distribution among the plant organs but especially on the species (Meneghelli et al., 2021). The hyperaccumulative *Brassicaceae*, *Caryophyllaceae*, *Polygonaceae*, and *Dichapetalaceae* can bioaccumulate up to 3,000 mg kg^–1^ dry weight of Zn (on average) (Broadley et al., 2007). In general, to support vital and metabolic functions, most plants need foliar Zn concentrations greater than 15-30 mg kg^–1^ dry weight, under which inhibition of photosynthesis and respiration rate, disruption of plasma membranes, increase in reactive oxygen species (ROS), and reduction in yield are observed (White and Broadley, 2011; Sharma T.R. et al., 2020; Buturi et al., 2021; Praharaj et al., 2021). However, in non-hyperaccumulative species, foliar concentrations of Zn over 100-700 mg kg^–1^ dry weight are toxic (White et al., 2018), causing growth reduction and yield suppression, chlorosis and leaf necrosis, reduced shoot and root development, reduced stomatal conductance and net carbon dioxide fixation, reduced and structural changes in chlorophyll, altered mitotic activity and membrane permeability, oxidative stress and lipid peroxidation, thus constraining biofortification programs (Tsonev and Cebola Lidon, 2012; Marichali et al., 2014). Although several authors have reported critical ranges of Zn in cabbage (74-1,201 mg kg^–1^), lettuce (20-60 mg kg^–1^), broccoli (117-1,666 mg kg^–1^), and leafy greens (up to 700 mg kg^–1^) (Maynard and Hochmuth, 2006; White and Broadley, 2011), to our knowledge, no research has studied the effects of Zn biofortification on basil (*Ocimum basilicum* L.). The scientific literature has not classified basil as either a hyperaccumulator or non-hyperaccumulator species. In any case, in light of the encouraging results obtained with Selenium and Iodine biofortification programs (Incrocci et al., 2019; Puccinelli et al., 2020; Puccinelli et al., 2021) we hypothesize that conditions dictated by hydroponics (floating raft system) and the use of biofortified nutrient solutions at different concentrations of Zinc (12.5, 25.0, 37.5, and 50 µM) would help to understand the relationships between Zinc and basil. Based on the above, our study aimed to evaluate the impact of biofortification on the yield, physiological responses, quality, and Zn bioaccumulation in two basil cultivars (Aroma 2 and Eleonora) grown in a floating raft system. The current work constitutes an important continuation of our earlier work (recently submitted for publication) that examines Zn biofortification of Genovese basil concerning the crop’s mineral profile and the implications of biofortification applications on estimated daily intake of adults and children.

## Materials and methods

### Experimental design and growth conditions

The experimental trial was conducted at the Department of Agriculture, Federico II University, (Portici, NA, Italy; 43° 10’ N; 14° 58’ E, 60 m a.s.l.) in an unheated greenhouse from May 3 to 26, 2021. Genovese basil seedlings (*Ocimum basilicum* L.) ‘Aroma 2’ (Fenix, Belpasso, CT, Italy) and ‘Eleonora’ (Enza Zaden, Enkhuizen, NL-NH, The Netherlands) were sown at a density of 317 pt m^–2^ on April 13, 2021 in peat and vermiculite (1:2 *v/v*) in 54-hole polystyrene trays (52 × 32 × 6 cm; volume 0.06 L) and grown in a floating raft system (FRS) in individual plastic trays filled with 35 L of nutrient solution (NS). As indicated by Ciriello et al. (2021a), a control nutrient solution with osmotic water was prepared using the following concentration of macro and micronutrients: 14 mM N-NO_3_^-^, 1.5 mM P, 1.75 mM S, 3.0 mM K, 4.5 mM Ca, 1.5 mM Mg, 1.0 mM NH_4_^+^, 15 MM Fe, 9 MM Mn, 1.6 MM Zn, 0.3 MM Cu, 20 MM B, and 0.3 MM Mo. The trial was carried out in a randomized design with three replicates in a factorial arrangement (2 × 5), with two basil cultivars (Aroma 2 and Eleonora) and four biofortification treatments plus control. The latter consisted of four doses of Zn (12.5, 25, 37.5, and 50 MM) using ZnSO_4_ × 7H_2_O (Sigma-Aldrich, St. Louis, MO, USA) as a Zn source in the nutrient solution. Each experimental unit consisted of 54 plants. Biofortified nutrient solutions were provided twenty days after planting (at the phenological stage of two true leaves). The biofortified treatment lasted for 23 days. Plants were grown under natural light conditions. During the growing cycle, temperature and relative air humidity were recorded continuously with an interval of 10 minutes with dedicated WatchDog A150 dataloggers (Spectrum Technologies Inc., Aurora, IL, USA) placed at canopy level. Specifically, the day/night average air temperature and relative humidity were 27/18°C and 50/70%, respectively.

### Sampling and determination of biometric and yield parameters

Before flowering (43 days after sowing), twenty plants per replicate were sampled for the determination of height (cm), number of leaves, and fresh biomass (g plant^–1^). Leaf area was quantified using ImageJ v1.52a software (U.S. National Institutes of Health of the United States, Bethesda, USA). The epigeal parts of each plant were dried in a ventilated oven at 70°C for 3 days to determine the dry biomass of the shoots and roots (g plant^–1^) and their percent ratio. The dry matter (%) of the shoots was calculated as follows:


$$\frac{Shoots\;dry\;biomass}{Shoots\;fresh\;biomass}\times 100$$

The dry plant material was ground and sieved using an MF10.1 Wiley laboratory mill equipped with an MF0.5 sieve (IKA, Staufen im Breisgau, BW, Germany) for mineral concentration determination. A representative plant sample was collected for each experimental unit and stored in liquid nitrogen for further qualitative analysis.

### Leaf color assessment

Leaf color assessment according to human vision was performed using the CIELab color space defined by the CIE (International Commission on Illumination), which separates greyscale (L*) information more clearly from color (a* and b*) information. The color coordinates were measured on twenty young fully expanded leaves per replicate using a Minolta CR-300 colorimeter (Minolta Co. Ltd, Osaka, Japan). Chroma and hue angle were calculated from the equations reported by Ciriello et al. (2021a).

### Physiological parameters, SPAD index, and pigments measurement

Leaf gas exchange measurements were performed with a Li-6400 hand-held analyzer (LI-COR Biosciences, Lincoln, NE, USA). The measured parameters of interest were the net assimilation of CO_2_ (ACO_2_), the transpiration rate (E), and the stomatal conductance (gs). Relative humidity (RH) and CO_2_ concentration of the leaf gas exchange analyzer were set at ambient values, while photosynthetically active radiation (PAR) and airflow rate were constant at 2,000 Mmol m^–2^ s^–1^ and 500 mL s^–1^, respectively. Chlorophyll fluorescence (Fv/Fm) measurements were made using a fluorometer Fv/Fm meter (Opti-Sciences, Hudson, NH, USA). The SPAD index was assessed using a Minolta SPAD-502 chlorophyll meter (Minolta Camera Co. Ltd., Osaka, Japan). All physiological measurements were performed between 09:30 and 12:00 am on five fully expanded healthy young leaves for each replicate.

Chlorophyll a and b concentrations were determined by UV-Vis spectrophotometry (ONDA V-10 Plus, Giorgio Bormac Srl, Carpi, Italy) with an absorbance of 647 and 664, respectively, as described by Wellburn (1994). Total chlorophyll was calculated as chlorophyll a + chlorophyll b and was expressed as mg g^–1^ fresh weight (fw).

The *β*-carotene and lutein concentrations were quantified by high-performance liquid chromatography with diode array detection (HPLC-DAD) after extraction according to Salomon et al. (2020). External standards of β-carotene and lutein (Sigma-Aldrich, Milan, Italy) were used to create the respective calibration curves. The results were expressed as µg g^–1^ dw.

### Determination of Zn concentration

According to the method described by Volpe et al. (2015) the Zn concentration in basil [Mg g^–1^ dry weight (dw)] was determined by an inductively coupled plasma mass spectrometer (ICP-OES Spectroblue, Spectro Ametek, Berwyn, PA, USA) after digestion with a mixture of HCl (37%) and HNO_3_ (65%) (3:9, *v/v*). An appropriate calibration curve was prepared using a standard solution with 1.0 to 100 Mg L^–1^ Zn concentrations.

### ABTS, DPPH, and FRAP antioxidant activities determination

The antioxidant activities ABTS^+^ (2,2′-azinobis-(3-ethylbenzothiazoline-6-sulfonate), DPPH (2,2-diphenyl-1-picrylhydrazyl), and FRAP (ferric reduction antioxidant potency) were determined by UV-VIS spectrophotometry (Shimadzu, Japan) according to the protocols described by Formisano et al. (2021). Results were expressed as mmol Trolox equivalents kg^–1^ dw. Phenolic Concentration Determination

One hundred milligrams of freeze-dried basil were used to quantify and determine polyphenols using a UHPLC system (Thermo Fisher Scientific, Waltham, MA, USA) equipped with a thermo-stated column (T=25°C, 100 × 2.1 mm, Kinetex 1.7 µm biphenyl, Phenomenex, Torrance, CA, USA) and a quaternary pump (Ultimate 3000, Dionex, Sunnyvale, CA, USA). Mass spectrometry analysis was facilitated by a Q Exactive Orbitrap LC-MS/MS system (Thermo Fisher Scientific, Waltham, MA, USA). Phenolic compound sampling was performed according to the protocol detailed by Pannico et al. (2020). The accuracy and calibration of the instruments used were set and checked using a mixture of reference standards (Thermo Fisher Scientific, Waltham, MA, USA). Data processing and analysis were performed using Xcalibur software, version 3.0.63 (Thermo Fisher Scientific, Waltham, MA, USA), and the results were expressed as µg g^–1^ dw.

### Statistics

Data were subjected to analysis of variance (ANOVA) and means for cultivar treatment (CV) were compared by the Student’s t-test, whereas the means for the biofortification treatment (Zn) and the two-way interaction (CV × Zn) were compared using the Tukey-Kramer HSD test at the *p*< 0.05 level. SPSS 20 software package (IBM Corp., Armonk, NY, USA) was used. Data represent mean ± standard error of 3 replicates (*n* = 3).

## Results

### Biometric and yield parameters

Plant height, number of leaves, fresh biomass, total dry biomass, and dry matter content for the control treatment were higher for ‘Aroma 2’ than ‘Eleonora’ (**Table 1**). On the other hand, the plant root dry weight of ‘Eleonora’ was higher than ‘Aroma 2’. Significant Cultivar × Zn biofortification interaction was observed for all the parameters presented in **Table 1**, since Zn biofortification treatments did not always have the same effect on both cultivars. In particular, while both cultivars’ plant height was negatively affected by only one Zn solution treatment compared to the control, this treatment differed between ‘Aroma 2’ (12.5 MM) and ‘Eleonora’ (37.5 µM). Leaf area was reduced on average by 17.1% in ‘Aroma 2’ and by 25.2% in ‘Eleonora’. It is noteworthy that the 25 MM Zn treatment did not have a significant effect on the leaf area of ‘Aroma 2’. In addition, the plant leaf number did not appear to be as affected by Zn concentration treatments in ‘Aroma 2’ compared to ‘Eleonora’, in which it decreased analogously to increasing Zn concentration. Similarly, while ‘Eleonora’ fresh biomass declined almost uniformly with increasing Zn concentration in the nutrient solution, ‘Aroma 2’ fresh biomass was not affected by all Zn treatments. On the other hand, while Zn treatments affected both cultivars’ total dry biomass negatively, Zn treatments overall reduced the dry biomass of ‘Aroma 2’ almost double (19%) that of ‘Eleonora’ (10%), compared in either case to the control. Cultivar differentiation with Zn biofortification was also observed in relation to dry matter content. The latter increased in ‘Eleonora’ in comparison to the control with all Zn concentration levels applied, while in ‘Aroma 2’ an increase was observed only after the application of 50.0 µM Zn in the nutrient solution. However, the application of the highest Zn (50 µM) concentration in the nutrient solution resulted in the highest content of dry matter in both cultivars. Dissimilar was also the behavior of the two cultivars concerning root dry weight, as ‘Aroma 2’ root dry weight decreased only with the application of 12.5 µM Zn in the nutrient solution, while all Zn treatments, except for 50 µM Zn, reduced the root dry weight of ‘Eleonora’ compared to the Control.

**Table 1:** Analysis of variance and mean comparisons for height, leaf number, leaf area, plant fresh biomass, total dry biomass, root dry weight, and dry matter of Aroma 2 and Eleonora basil cultivars grown hydroponically under different Zn treatments: 0 = Control; 12.5; 25; 37.5; 50 µmol of Zn].

Treatment	Height		Leaf number		Leaf area		Plant fresh biomass		Total dry biomass		Root dry weight		Dry matter	
	cm		n°		cm^2^		g plant^–1^						%	
Cultivar (CV)														
Aroma 2	40.02 ± 0.32	a	41.65 ± 0.91	a	405.524 ± 9.017		23.41 ± 0.22	a	2.31 ± 0.06	a	0.221 ± 0.003	b	10.4 ± 0.09	a
Eleonora	35.48 ± 0.28	b	35.98 ± 1.24	b	393.971 ± 14.935		19.17 ± 0.53	b	1.86 ± 0.03	b	0.249 ± 0.007	a	9.64 ± 0.13	b
Zinc (Zn)														
Control	38.01 ± 1.02	a	45.92 ± 0.86	a	477.471 ± 10.989	a	23.23 ± 0.66	a	2.38 ± 0.16	a	0.256 ± 0.012	a	9.52 ± 0.24	d
12.5 µM	38.40 ± 1.39	a	38.88 ± 1.74	b	398.609 ± 5.555	b	21.78 ± 0.89	b	1.96 ± 0.08	c	0.218 ± 0.004	c	9.99 ± 0.17	b
25 µM	36.94 ± 0.57	b	38.60 ± 0.80	b	398.794 ± 10.659	b	21.74 ± 0.78	b	2.01 ± 0.09	c	0.213 ± 0.003	c	9.74 ± 0.16	c
37.5 µM	36.59 ± 1.27	b	35.98 ± 1.36	c	360.941 ± 5.043	c	20.52 ± 1.09	c	1.97 ± 0.08	c	0.236 ± 0.004	b	10.17 ± 0.18	b
50 µM	38.80 ± 0.90	a	34.69 ± 1.83	c	362.924 ± 9.613	c	19.19 ± 1.39	d	2.12 ± 0.10	b	0.251 ± 0.010	a	10.67 ± 0.13	a
CV × Zn														
Aroma 2 × Control	40.27 ± 0.33	ab	47.67 ± 0.36	a	461.225 ± 8.046	ab	24.68 ± 0.20	a	2.73 ± 0.01	a	0.229 ± 0.002	bc	10.06 ± 0.07	de
Aroma2 × 12.5	41.46 ± 0.16	a	42.76 ± 0.17	b	397.804 ± 5.086	cd	23.74 ± 0.13	ab	2.14 ± 0.02	c	0.212 ± 0.002	cd	10.36 ± 0.01	bcd
Aroma2 × 25	38.19 ± 0.19	c	40.25 ± 0.25	c	419.435 ± 3.633	bc	23.44 ± 0.08	bc	2.21 ± 0.03	c	0.205 ± 0.003	d	10.08 ± 0.12	cde
Aroma2 × 37.5	39.40 ± 0.30	bc	38.83 ± 0.98	cd	366.158 ± 4.701	de	22.92 ± 0.06	bcd	2.15 ± 0.03	c	0.228 ± 0.004	bc	10.56 ± 0.04	b
Aroma2 × 50	40.79 ± 0.02	a	38.75 ± 0.47	cd	383.000 ± 3.704	cde	22.28 ± 0.09	cd	2.34 ± 0.01	b	0.232 ± 0.003	bc	10.92 ± 0.14	a
Eleonora × Control	35.75 ± 0.10	de	44.17 ± 0.68	b	493.718 ± 16.586	a	21.78 ± 0.11	d	2.03 ± 0.03	d	0.282 ± 0.006	a	8.98 ± 0.03	h
Eleonora × 12.5	35.33 ± 0.43	e	35.00 ± 0.17	ef	399.415 ± 11.303	cd	19.82 ± 0.30	e	1.78 ± 0.01	f	0.224 ± 0.001	bcd	9.62 ± 0.02	fg
Eleonora × 25	35.69 ± 0.10	de	36.96 ± 0.65	de	378.153 ± 11.349	cde	20.04 ± 0.35	e	1.81 ± 0.02	ef	0.222 ± 0.004	cd	9.39 ± 0.03	g
Eleonora × 37.5	33.79 ± 0.40	f	33.13 ± 0.38	fg	355.724 ± 8.823	de	18.12 ± 0.37	f	1.79 ± 0.01	f	0.245 ± 0.002	b	9.78 ± 0.02	ef
Eleonora × 50	36.81 ± 0.29	d	30.63 ± 0.14	g	342.849 ± 6.728	e	16.09 ± 0.33	g	1.90 ± 0.01	e	0.271 ± 0.010	a	10.42 ± 0.08	bc
Significance														
CV	***		***		ns		***		***		***		***	
Zn	***		***		***		***		***		***		***	
CV × Zn	***		***		**		***		***		***		*	

* Significant effect at the 0.05 level, ** 0,01 level, *** 0.001 level, ns, non-significant effect. Data represent means ± standard error of 3 replicates (n=3). Treatment means within each column followed by different letters denote significant differences (P < 0.05) according to Tukey-Kramer HSD test.

### Colorimetric parameters

Significant CV × Zn interactions were observed for all leaf colorimetric parameters analyzed (L*, a*, C* and h°; **Table 2**), which indicates a cultivar-dependent response to Zn biofortification in terms of leaf colorimetry. Leaf coloration of ‘Aroma 2’ was darker in the control compared to all Zn biofortification treatments, whereas ‘Eleonora’ was non-responsive at any level of Zn biofortification. The intensity of green color was minimally reduced in ‘Aroma 2’ only in response to the 50 MM Zn application, as denoted by lower negative values of a*; contrarily, all Zn treatments increased the intensity of green color in ‘Eleonora’ compared to the control, with saturation observed at 25 MM Zn or higher. Cultivar behavior was also dissimilar with respect to hue angle (h°). In ‘Aroma 2’, hue angle decreased at Zn level 25.0 MM or higher, denoting a tendency for yellower hue, whereas in ‘Eleonora’ all Zn levels except 50.0 MM resulted in greener hue than the control.

**Table 2:** Analysis of variance and mean comparisons for leaf colorimetric components L*, a*, b*, Chroma, and Hue angle of Aroma 2 and Eleonora basil cultivars grown hydroponically under different Zn treatments: 0 = Control; 12.5; 25; 37.5; 50 µmol of Zn].

Treatment	L*		a*		b*		Chroma		Hue Angle (°)	
Cultivar (CV)										
Aroma 2	44.51 ± 0.29		–9.22 ± 0.15	b	21.99 ± 0.34	b	24.00 ± 0.31	b	112.75 ± 0.40	a
Eleonora	44.64 ± 0.08		–8.84 ± 0.29	a	24.61 ± 0.65	a	26.43 ± 0.73	a	110.62 ± 0.19	b
Zinc (Zn)										
Control	43.85 ± 0.48	c	–8.16 ± 0.53	a	20.09 ± 0.27	d	21.71 ± 0.39	c	112.01 ± 1.14	a
12.5 µM	44.04 ± 0.13	c	–8.97 ± 0.29	b	23.66 ± 0.93	bc	25.75 ± 0.98	ab	112.67 ± 0.69	a
25 µM	45.00 ± 0.20	ab	–9.54 ± 0.06	c	24.28 ± 0.61	ab	26.38 ± 0.68	ab	112.01 ± 0.43	a
37.5 µM	45.23 ± 0.15	a	–9.47 ± 0.04	c	24.85 ± 0.45	a	26.64 ± 0.46	a	110.96 ± 0.10	b
50 µM	44.76 ± 0.17	b	–9.01 ± 0.39	b	23.63 ± 1.08	c	25.58 ± 0.98	b	110.78 ± 0.16	b
CV × Zn										
Aroma 2 × Control	42.79 ± 0.18	f	–9.34 ± 0.06	c	20.34 ± 0.07	de	22.39 ± 0.15	de	114.52 ± 0.24	a
Aroma2 × 12.5	43.77 ± 0.10	e	–9.60 ± 0.03	c	21.59 ± 0.04	c	23.64 ± 0.12	cd	114.13 ± 0.41	ab
Aroma2 × 25	45.41 ± 0.06	a	–9.60 ± 0.09	c	22.95 ± 0.26	b	24.91 ± 0.3	bc	112.92 ± 0.17	b
Aroma2 × 37.5	45.46 ± 0.25	a	–9.41 ± 0.08	c	23.86 ± 0.12	b	25.66 ± 0.08	b	111.13 ± 0.11	c
Aroma2 × 50	45.12 ± 0.12	ab	–8.14 ± 0.17	b	21.22 ± 0.2	cd	23.41 ± 0.05	cd	111.05 ± 0.16	c
Eleonora × Control	44.91 ± 0.01	abcd	–6.99 ± 0.15	a	19.84 ± 0.54	e	21.04 ± 0.55	e	109.49 ± 0.37	d
Eleonora × 12.5	44.32 ± 0.05	de	–8.35 ± 0.20	b	25.73 ± 0.12	a	27.87 ± 0.53	a	111.2 ± 0.24	c
Eleonora × 25	44.59 ± 0.16	bcd	–9.48 ± 0.06	c	25.62 ± 0.16	a	27.85 ± 0.20	a	111.1 ± 0.25	c
Eleonora × 37.5	45.00 ± 0.03	abc	–9.53 ± 0.01	c	25.84 ± 0.11	a	27.61 ± 0.35	a	110.79 ± 0.08	c
Eleonora × 50	44.40 ± 0.01	cd	–9.87 ± 0.06	c	26.03 ± 0.05	a	27.75 ± 0.17	a	110.5 ± 0.19	cd
Significance										
CV	n.s.		***		***		***		***	
Zn	***		***		***		***		***	
CV × Zn	***		***		***		***		***	

***Significant effect at the 0.001 level, ns, non-significant effect. Data represent means ± standard error of 3 replicates (n=3). Treatment means within each column followed by different letters denote significant differences (P< 0.05) according to Tukey-Kramer HSD test.

### Physiological parameters and pigments

Cultivar physiology under control conditions was similar for all the parameters examined, except for CO_2_ assimilation rate (ACO_2_), since ‘Aroma 2’ exhibited a higher ACO_2_ than ‘Eleonora’ (**Table 3**). In general, adding Zn to the nutrient solution appeared to stress both cultivars based on their physiological parameters. However, cultivar response to Zn treatment levels was not uniform, as indicated by the significant CV × Zn interaction. The interaction had an impact on all physiological attributes except for ACO_2_, which was reduced on average by 13% for both cultivars under supplemental Zn treatments in the nutrient solution. Stomatal conductance was reduced on average by 16.3% and 21.3% for ‘Aroma 2’ and ‘Eleonora, respectively. The lowest Zn treatment did not have an effect on ‘Eleonora’ stomatal function. Although transpiration rate (E) of ‘Aroma 2’ was not affected, transpiration of ‘Eleonora’ was reduced on average by 19.9% for treatments exceeding 12.5 MM Zn in the nutrient solution. Zinc treatments decreased SPAD index of ‘Eleonora’ by 12.3% on average, while only the intermediate Zn treatments (25 and 37.5 MM) reduced the chlorophyll concentration of ‘Aroma 2’ by 4.3%, on average. Also. Zn treatments negatively affected the maximum quantum yield of photosystem II (Fv/Fm) of both cultivars, namely by 4.2% and 3.9% on average, for ‘Aroma 2’ and ‘Eleonora’, respectively. However, while ‘Eleonora’ Fv/Fm appeared to decrease proportionally with increasing Zn concentration, ‘Aroma 2’ Fv/Fm decreased up to 25MM concentration and showed no further change thereafter.

**Table 3:** Analysis of variance and mean comparisons for net CO_2_ assimilation rate (ACO_2_), stomatal conductance (gs*)*, transpiration (E), SPAD index, Fv/Fm, total chlorophyll, and carotenoids of Aroma 2 and Eleonora basil cultivars grown hydroponically under different Zn treatments: 0 = Control; 12.5; 25; 37.5; 50 µmol of Zn].

Treatment	ACO_2_		gs		E		SPAD index		Fv/Fm		Total Chlorophyll		Lutein		*β*-Carotene	
	µmol CO_2_ m^–2^ s^–1^		mol H_2_O m^–2^ s^–1^		mol H_2_O m^–2^ s^–1^						mg g^–1^ fw		µg g^–1^ dw			
Cultivar (CV)																
Aroma 2	19.16 ± 0.38	a	0.212 ± 0.01	a	4.12 ± 0.06		37.43 ± 0.18	a	0.796 ± 0.004	b	1.73 ± 0.04		992.35 ± 34.06	a	410.71 ± 8.30	a
Eleonora	18.54 ± 0.35	b	0.190 ± 0.01	b	4.06 ± 0.12		34.28 ± 0.62	b	0.803 ± 0.005	a	1.69 ± 0.07		890.77 ± 21.70	b	401.09 ± 11.51	b
Zinc (Zn)																
Control	21.03 ± 0.28	a	0.231 ± 0.01	a	4.57 ± 0.09	a	38.31 ± 0.24	a	0.826 ± 0.003	a	2.03 ± 0.05	a	907.24 ± 12.70	c	370.14 ± 4.00	d
12.5 µM	19.73 ± 0.19	b	0.213 ± 0.01	b	4.25 ± 0.09	ab	36.02 ± 0.58	b	0.809 ± 0.003		1.76 ± 0.01	b	838.14 ± 33.38	c	394.94 ± 5.82	c
25 µM	17.82 ± 0.21	c	0.188 ± 0.01	c	3.82 ± 0.10	c	35.78 ± 0.53	b	0.794 ± 0.004	c	1.70 ± 0.02	b	879.66 ± 12.43	c	388.88 ± 3.47	c
37.5 µM	18.08 ± 0.24	c	0.187 ± 0.01	c	3.93 ± 0.11	bc	34.78 ± 0.98	c	0.788 ± 0.003	c	1.57 ± 0.02	c	987.06 ± 44.81	b	419.93 ± 17.66	b
50 µM	17.59 ± 0.15	c	0.184 ± 0.01	c	3.88 ± 0.10	c	34.40 ± 1.38	c	0.779 ± 0.002	d	1.47 ± 0.03	d	1095.7 ± 47.46	a	455.60 ± 13.69	a
CV × Zn																
Aroma 2 × Control	21.38 ± 0.50		0.243 ± 0.01	a	4.47 ± 0.03	ab	38.60 ± 0.44	a	0.824 ± 0.002	ab	1.93 ± 0.02	b	886.18 ± 12.18	d	377.65 ± 4.34	de
Aroma2 × 12.5	20.04 ± 0.23		0.211 ± 0.01	b	4.08 ± 0.01	bc	37.24 ± 0.20	ab	0.804 ± 0.002	cd	1.78 ± 0.02	c	911.11 ± 7.97	cd	407.27 ± 4.10	cd
Aroma2 × 25	18.26 ± 0.16		0.203 ± 0.01	b	4.03 ± 0.03	bc	36.94 ± 0.07	b	0.786 ± 0.001	ef	1.72 ± 0.01	cd	898.72 ± 18.96	cd	381.86 ± 1.97	de
Aroma2 × 37.5	18.52 ± 0.16		0.207 ± 0.01	b	4.07 ± 0.15	bc	36.97 ± 0.01	b	0.783 ± 0.001	ef	1.61 ± 0.02	de	1080.21 ± 36.33	ab	458.09 ± 10.11	ab
Aroma2 × 50	17.61 ± 0.28		0.194 ± 0.01	bc	3.93 ± 0.06	bc	37.40 ± 0.18	ab	0.783 ± 0.002	ef	1.54 ± 0.02	e	1185.52 ± 53.64	a	428.65 ± 4.09	bc
Eleonora × Control	20.68 ± 0.16		0.219 ± 0.01	ab	4.67 ± 0.18	a	38.02 ± 0.07	ab	0.829 ± 0.004	a	2.13 ± 0.04	a	928.30 ± 14.63	cd	362.63 ± 2.13	e
Eleonora × 12.5	19.42 ± 0.19		0.214 ± 0.01	b	4.42 ± 0.10	ab	34.80 ± 0.42	c	0.813 ± 0.004	bc	1.75 ± 0.02	c	765.17 ± 13.50	e	382.61 ± 0.78	de
Eleonora × 25	17.39 ± 0.13		0.173 ± 0.01	cd	3.61 ± 0.03	c	34.61 ± 0.18	c	0.803 ± 0.002	cd	1.69 ± 0.04	cd	860.60 ± 7.07	de	395.89 ± 2.68	d
Eleonora × 37.5	17.64 ± 0.25		0.168 ± 0.01	d	3.78 ± 0.14	c	32.59 ± 0.17	d	0.794 ± 0.002	de	1.53 ± 0.02	e	893.91 ± 6.66	cd	381.77 ± 0.79	de
Eleonora × 50	17.58 ± 0.19		0.174 ± 0.01	cd	3.83 ± 0.20	c	31.39 ± 0.72	d	0.777 ± 0.003	f	1.40 ± 0.01	f	1005.88 ± 17.83	bc	482.55 ± 13.92	a
Significance																
CV	***		***		n.s.		***		***		n.s.		***		*	
Zn	***		***		***		***		***		***		***		***	
CV × Zn	n.s.		**		*		***		**		***		***		***	

* Significant effect at the 0.05 level, ** 0.01 level, *** 0.001 level, ns, non-significant effect. Data represent means ± standard error of 3 replicates (n=3). Treatment means within each column followed by different letters denote significant differences (P < 0.05) according to Tukey-Kramer HSD test.

Under control conditions, lutein and *β* -carotene values were higher in ‘Aroma 2’ than ‘Eleonora’, although the latter had a higher total chlorophyll concentration (**Table 3**). Zn application had a significant effect on all basil plant pigments when compared to the control. However, CV × Zn interaction was observed for all basil pigments since Zn treatments did not affect uniformly the two cultivars. For instance, the addition of Zn to the nutrient solution had a much greater effect overall on ‘Eleonora’ plant total chlorophyll concentration, which decreased on average by 79.1% more than that of ‘Aroma 2’. Furthermore, the lutein concentration of ‘Eleonora’ was reduced only when plants were treated at the lowest Zn concentration (12.5 µM), whereas the lutein concentration of ‘Aroma 2’ increased (on average by 27.8%) only when plants were exposed to the highest Zn treatments (37.5 - 50 µM). Finally, the *β*-carotene concentration of the two cultivars increased only when plants of ‘Aroma 2’ were exposed to the highest Zn treatments, 37.5 and 50 µM, and when ‘Eleonora’ plants were exposed to 25 and 50 MM Zn.

### Plant root and shoot Zn accumulation

**Figure 1:**
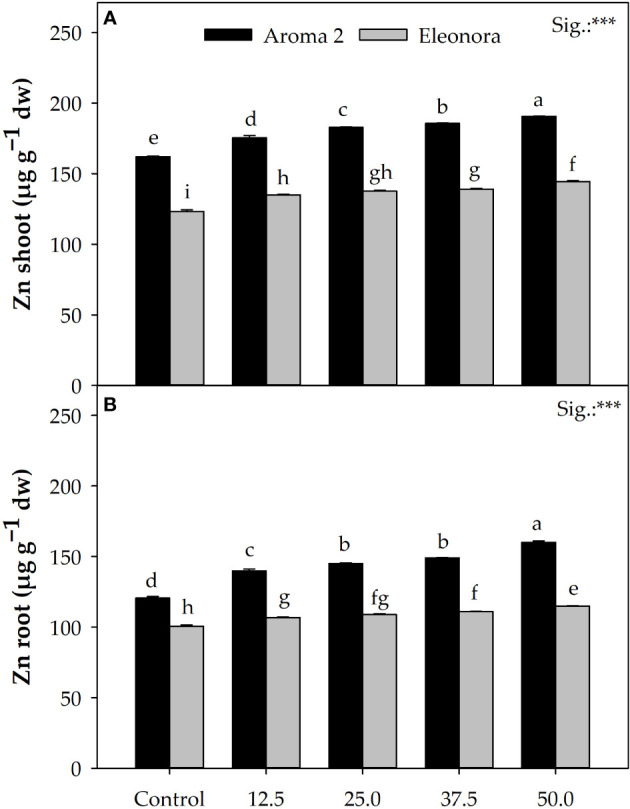
Interaction plots among Cultivar × Zn treatment for Zn accumulation in shoots **(A)** and roots **(B)** of ‘Aroma 2’ and ‘Eleonora’ basil cultivars grown hydroponically under different Zn treatments (Zn): 0 = Control; 12.5; 25; 37.5; 50 µM of Zn. Data represent means of 3 replicates (n=3). *** significant effect at the 0.001 level. Different letters denote significant differences (P ≤ 0.05) according to Tukey-Kramer HSD test.

Significant cultivar differentiation was observed with respect to Zn accumulation in the root and shoot of plants as ‘Aroma 2’ grown in the control solution accumulated on average more Zn in their roots (31.4%) and shoots (19.9%) than the ‘Eleonora’ control plants. Increasing Zn concentration in the nutrient solution resulted in both cultivars showing a relative increase in root and shoot Zn concentration. Overall, supplementing the nutrient solution with Zn increased root Zn concentration in ‘Aroma 2’ by 13.4% and ‘Eleonora’ by 12.8%, when compared to the control. Notable was also the differentiation in Zn concentration by the shoots of the two cultivars compared to the control, as Zn accumulation in the shoots increased on average by 23.1% in ‘Aroma 2’ compared to 9.7% in ‘Eleonora’. This disproportional Zn accumulation potential in the shoots of the two cultivars was manifested as significant CV × Zn interaction. In fact, Zn addition to the nutrient solution had a greater effect on ‘Aroma 2’ plant shoot Zn levels compared to the control, which was overall approximately 138.8% higher than that of ‘Eleonora’ (**Figure 1A**). Interaction was observed also concerning Zn root levels as the behavior of the two cultivars differed in terms of root Zn concentration following the gradual Zn concentration increase in the nutrient solution. Specifically, raising Zn solution concentration from 12.5 to 25 MM increased root Zn accumulation of ‘Aroma 2’ by 116.7% more than that of ‘Eleonora’. Conversely, the shift from 37.5 to 50 MM of Zn in the nutrient solution increased ‘Eleonora’ root Zn upload by 43.7% more than that of ‘Aroma 2’ (**Figure 1B**).

### Antioxidant activity

The antioxidant activity as assessed by the DPPH, FRAP, and ABTS assays, were higher in the control treatment of ‘Aroma 2’ than that of ‘Eleonora’. Both cultivars’ antioxidant activity (DPPH, FRAP, and ABTS) was altered by supplemental Zn in the nutrient solution (**Table 4**). The antioxidant activities determined by DPPH, FRAP, and ABTS assays were all significantly affected by CV × Zn interaction as the addition of Zn affected the antioxidant activity of ‘Eleonora’ to a much greater extent than that of ‘Aroma 2’, moreover several Zn levels did not have the same effect on the two cultivars. Overall, the increase of supplemental Zn from 12.5 to 50 MM increased the antioxidant activity of ‘Eleonora’ by 159.7%, 229%, and 188.8% more than that recorded for ‘Aroma 2’ in terms of DPPH, FRAP, and ABTS, respectively. Notable was the fact that the DPPH activity of ‘Aroma 2’ was reduced even by the lowest Zn treatment while the 12.5 and 25 MM Zn levels did not have any effect on the same cultivar’s ABTS activity.

**Table 4:** Analysis of variance and mean comparisons for DPPH, FRAP, and ABTS antioxidant activities of Aroma 2 and Eleonora basil cultivars grown hydroponically under different Zn treatments: 0 = Control; 12.5; 25; 37.5; 50 µmol of Zn].

Treatment	DPPH		FRAP		ABTS	
	mmol trolox kg^–1^ dw				
Cultivar (CV)						
Aroma 2	218.23 ± 9.60	a	186.29 ± 4.34	a	233.87 ± 3.43	a
Eleonora	206.34 ± 11.53	b	154.18 ± 6.83	b	209.67 ± 9.70	b
Zinc (Zn)						
Control	162.05 ± 13.07	e	140.33 ± 12.27	e	192.61 ± 13.01	e
12.5 µM	188.93 ± 7.00	d	165.15 ± 5.70	c	208.23 ± 7.80	d
25 µM	206.62 ± 1.28	c	159.85 ± 7.35	d	221.65 ± 2.26	c
37.5 µM	234.94 ± 4.79	b	189.07 ± 4.59	b	228.23 ± 11.47	b
50 µM	268.90 ± 1.61	a	196.77 ± 6.39	a	258.13 ± 6.40	a
CV × Zn						
Aroma 2 × Control	191.23 ± 0.51	e	167.70 ± 1.33	d	221.66 ± 1.31	c
Aroma2 × 12.5	173.51 ± 1.21	f	177.24 ± 1.50	c	225.40 ± 3.03	c
Aroma2 × 25	208.98 ± 0.64	d	176.27 ± 0.48	c	224.90 ± 2.02	c
Aroma2 × 37.5	245.45 ± 0.78	b	199.27 ± 0.90	b	253.51 ± 1.09	b
Aroma2 × 50	271.98 ± 1.66	a	210.98 ± 0.94	a	243.90 ± 0.94	b
Eleonora × Control	132.87 ± 1.68	g	112.97 ± 1.34	g	163.55 ± 0.36	f
Eleonora × 12.5	204.34 ± 2.36	d	153.06 ± 3.74	e	191.07 ± 0.26	e
Eleonora × 25	204.25 ± 1.48	d	143.43 ± 0.51	f	218.41 ± 3.30	c
Eleonora × 37.5	224.42 ± 1.94	c	178.88 ± 0.80	c	202.96 ± 4.22	d
Eleonora × 50	265.81 ± 0.87	a	182.55 ± 1.04	c	272.35 ± 1.37	a
Significance						
CV	***		***		***	
Zn	***		***		***	
CV × Zn	***		***		***	

*** Significant effect 0.001 level. Data represent means ± standard error of 3 replicates (n=3). Treatment means within each column followed by different letters denote significant differences (P< 0.05) according to Tukey-Kramer HSD test.

### Phenolic acids

Ten phenolic acids were identified in both basil cultivars (**Table 5**). Chicoric acid, was the most abundant phenolic acid in the control solution for both cultivars with a value of 4872.7 Mg g^−1^ dw and 4319 Mg g^−1^ dw for ‘Aroma 2’ and ‘Eleonora’ respectively, while rosmarinic acid was the second most abundant phenolic acid (523.8 Mg g^−1^ dw for ‘Aroma 2’ and 358.5 Mg g^−1^ dw for ‘Eleonora’). The ranking of the remaining phenolic acids was also similar in the two cultivars except for salvianic and caffeic acid. Salvianic and caffeic acids ranked 7^th^ and 9^th^ for ‘Aroma 2’, while it was the other way around for ‘Eleonora’. Under control conditions, ‘Aroma 2’ total phenolic acids concentration was higher by 12.8% than ‘Eleonora’, since half of the individual phenolic acids concentration (rosmarinic acid, salvianolic acid A, salvianolic acid K, chlorogenic acid, and salvianic acid A) was higher in ‘Aroma 2’ than in ‘Eleonora’. No differences among the two cultivars were observed for the rest of the phenolic acids (caftaric acid, caffeic acid, feruloyl tartaric acid, salvianolic acid L, and cichoric acid) in the control solution. Zinc additional quantity to the nutrient solution altered the individual phenolic acids concentration and the total phenolic concentration of both cultivars. An exception was recorded for ‘Eleonora’ salvianolic acid L concentration which was not affected by Zn treatments. The effect of Zinc, though, was subjected to significant CV × Zn interaction for all the individual phenolic acids and for the total phenolic acids concentration. In all the phenolic profile parameters examined, interaction was significant because Zn treatments did not have the same effect on the two cultivars. For example, all Zn levels influenced the total phenolic concentration of ‘Eleonora’ and ‘Aroma 2’, except the lowest Zn treatment which had no effect on ‘Aroma 2’. Furthermore, the addition of Zn to the nutrient solution overall, had a much greater effect on ‘Eleonora’ plant total phenolic concentration as the latter increased by 47.8% more than that of the ‘Aroma 2’ plants, compared to their respective controls. The CV × Zn interaction for chicoric acid concentration was also significant because while Zn levels up to 37.5 MM appeared to affect the two cultivars similarly, the highest Zn level (50 MM) increased the chicoric acid of ‘Aroma 2’ by 47.4% more than ‘Eleonora’, compared to the control. Regarding the rest of the phenolic acids and depending on the cultivar, some Zn levels had a positive or negative effect on one or the other or both cultivars while others had no effect at all. Specifically, certain levels of Zn (what is presented next in parentheses is the average value) increased the concentration compared to the control of salvianic acid A (‘Aroma 2’= 27.4%, ‘Eleonora’= 120.3%), caftaric acid (‘Aroma 2’= 20.4%, ‘Eleonora’= 22.1%), caffeic acid (‘Eleonora’= 42.3%), chlorogenic acid (‘Eleonora’= 13.2%), feruloyl tartaric acid (‘Aroma 2’= 15%, ‘Eleonora’= 24.9%), salvianolic acid K (‘Aroma 2’= 14.4%, ‘Eleonora’= 136%), salvianolic acid A (‘Aroma 2’= 21.1%, ‘Eleonora’= 149.9%), salvianolic acid L (‘Aroma 2’= 26.2%) and rosmarinic acid (‘Aroma 2’= 13.1%, ‘Eleonora’= 23.5%). Several Zn treatments had also a negative effect on ‘Aroma 2’ salvianic A (7.8%), caffeic (19%), chlorogenic (21.2%) and rosmarinic (13.1%) acids concentration.

**Table 5:** Analysis of variance and mean comparisons for phenolic profile of Aroma 2 and Eleonora basil cultivars grown hydroponically under different Zn treatments: 0 = Control; 12.5; 25; 37.5; 50 µmol of Zn].

Treatment	Salvianic acid A		Caftaric acid		Caffeic acid		Chlorogenicacid		Feruloyl tartaricacid			Salvianolic acid K			Salvianolicacid A		Salvianolicacid L		Rosmarinic acid		Cichoric acid		Total Phenolic		
	µg g^–1^ dw																								
Cultivar (CV)																									
Aroma	55.40 ± 2.41	a	51.12 ± 1.15		48.85 ± 1.51	b	9.59 ± 0.30	a		58.57 ± 0.96	b		224.84 ± 4.89	a	88.04 ± 3.23	a	102.77 ± 2.80	a	491.42 ± 8.62	a	4041.92 ± 60.70	a		5172.60 ± 69.59	a
Eleonora	11.04 ± 1.16	b	50.53 ± 1.40		61.27 ± 3.10	a	8.76 ± 0.16	b		63.43 ± 2.07	a		99.58 ± 9.27	b	81.24 ± 7.32	b	82.68 ± 2.84	b	426.00 ± 11.73	b	3900.97 ± 40.73	b		4785.51 ± 73.36	b
Zinc (Zn)																									
Control	28.50 ± 10.23	b	47.19 ± 0.81	c	54.06 ± 0.85	b	9.83 ± 0.64	a		52.60 ± 0.90	c		129.00 ± 37.27	d	56.13 ± 8.63	d	84.71 ± 6.90		441.12 ± 37.04	c	3692.51 ± 38.47	d		4595.83 ± 127.65	d
12.5 µM	27.78 ± 8.86	b	50.91 ± 0.63	b	45.21 ± 1.73	c	8.89 ± 0.18	b		64.26 ± 1.20	a		150.64 ± 26.86	c	85.77 ± 3.80	b	96.79 ± 1.44		432.83 ± 9.15	c	3913.77 ± 28.3	c		4876.83 ± 72.57	c
25 µM	29.27 ± 8.00	b	53.49 ± 2.48	a	51.09 ± 1.83	b	9.62 ± 0.28	a		62.20 ± 1.44	ab		156.94 ± 23.47	bc	77.79 ± 1.77	c	93.77 ± 5.35		440.51 ± 9.41	c	3978.15 ± 25.15	bc		4952.84 ± 55.01	c
37.5 µM	41.15 ± 13.14	a	48.80 ± 0.46	bc	60.92 ± 7.11	a	7.99 ± 0.09	c		59.42 ± 0.36	b		177.72 ± 31.33	ab	103.15 ± 3.46	a	97.29 ± 9.62		471.34 ± 12.63	b	4021.93 ± 26.43	b		5089.69 ± 86.17	b
50 µM	39.40 ± 9.41	a	53.73 ± 3.02	a	64.03 ± 4.29	a	9.54 ± 0.08	a		66.53 ± 3.86	a		196.75 ± 22.58	a	100.38 ± 8.12	a	91.09 ± 4.49		507.74 ± 8.62	a	4250.88 ± 79.96	a		5380.06 ± 103.97	a
CV × Zn																									
Aroma 2 × Control	51.38 ± 0.15	c	48.94 ± 0.38	bcd	55.33 ± 1.39	b	11.21 ± 0.40	a		52.29 ± 1.38	d		210.31 ± 0.14	b	75.32 ± 1.54	e	93.66 ± 4.74	bc	523.74 ± 3.18	a	3750.14 ± 63.13	de		4872.67 ± 65.06	def
Aroma2 × 12.5	47.59 ± 0.27	d	51.18 ± 1.38	b	41.52 ± 1.02	f	8.85 ± 0.36	cde		62.40 ± 0.51	bc		210.61 ± 0.15	b	93.94 ± 1.47	cd	97.01 ± 2.88	abc	449.47 ± 11.79	c	3974.62 ± 12.84	bc		5037.2 ± 22.85	cd
Aroma2 × 25	47.12 ± 0.96	d	58.94 ± 0.98	a	47.71 ± 1.90	def	10.22 ± 0.19	ab		60.76 ± 0.11	bc		209.38 ± 0.30	b	80.31 ± 0.61	e	103.91 ± 6.21	ab	460.94 ± 5.05	c	3985.45 ± 52.14	bc		5064.73 ± 49.08	c
Aroma2 × 37.5	70.53 ± 0.73	a	49.40 ± 0.72	bc	45.20 ± 1.43	ef	8.13 ± 0.10	de		59.08 ± 0.46	bcd		246.93 ± 4.23	a	108.05 ± 3.74	ab	118.18 ± 5.00	a	499.21 ± 1.95	ab	4075.55 ± 14.55	b		5280.26 ± 9.11	b
Aroma2 × 50	60.40 ± 1.30	b	47.14 ± 0.37	cd	54.49 ± 0.72	bc	9.53 ± 0.17	bc		58.30 ± 0.59	cd		246.98 ± 1.35	a	82.59 ± 1.85	de	101.10 ± 0.53	ab	523.75 ± 1.05	a	4423.84 ± 43.98	a		5608.12 ± 40.71	a
Eleonora × Control	5.62 ± 0.18	g	45.44 ± 0.32	d	52.79 ± 0.25	bcd	8.45 ± 0.08	de		52.91 ± 1.42	d		47.68 ± 18.21	e	36.94 ± 1.17	f	75.77 ± 11.66	c	358.51 ± 4.96	e	3634.88 ± 9.58	e		4319.00 ± 24.46	g
Eleonora × 12.5	7.97 ± 0.39	g	50.64 ± 0.08	bc	48.89 ± 0.57	cde	8.92 ± 0.17	cd		66.11 ± 1.89	b		90.67 ± 3.27	d	77.59 ± 1.79	e	96.57 ± 1.41	abc	416.19 ± 1.55	d	3852.91 ± 11.67	cd		4716.46 ± 9.44	f
Eleonora × 25	11.42 ± 0.31	f	48.05 ± 0.13	bcd	54.48 ± 1.31	bc	9.02 ± 0.04			63.64 ± 2.87	bc		104.51 ± 2.26	d	75.27 ± 2.99	e	83.63 ± 1.28	bc	420.09 ± 0.05	d	3970.85 ± 19.73	bc		4840.96 ± 14.25	ef
Eleonora × 37.5	11.77 ± 0.54	f	48.20 ± 0.43	bcd	76.64 ± 1.99	a	7.85 ± 0.08	e		59.75 ± 0.58	bcd		108.50 ± 9.92	d	98.25 ± 4.67	bc	76.40 ± 1.09	c	443.47 ± 4.11	cd	3968.3 ± 20.13	bc		4899.12 ± 26.93	de
Eleonora × 50	18.41 ± 0.22	e	60.33 ± 1.43	a	73.57 ± 0.75	a	9.56 ± 0.03	bc		74.75 ± 2.55	a		146.51 ± 5.00	c	118.17 ± 3.12	a	81.07 ± 0.57	bc	491.72 ± 10.67	b	4077.92 ± 10.84	b		5152.00 ± 19.47	bc
Significance																									
Cultivar (CV)	***		n.s.		***		***			***			***		***		***		***		***			***	
Zinc (Z)	***		***		***		***			***			***		***		n.s.		***		***			***	
CV × Z	***		***		***		***			***			**		***		**		***		***			***	

**Significant effect at the 0.01 level, *** 0.001 level, ns, non-significant effect. Data represent means ± standard error of 3 replicates (n=3). Treatment means within each column followed by different letters denote significant differences (P< 0.05) according to Tukey-Kramer HSD test.

## Discussion

In soilless growth systems, biofortification of the nutrient solution can augment the concentration, translocation and accumulation of trace elements in the edible plant organs due to the enhanced availability of trace elements, such as Zn, but also due to the absence of soil × root interaction (Wiesner-Reinhold et al., 2017). In this study, results showed that basil is characterized by high genetic variability in Zn accumulation capacity, as control plants of ‘Aroma 2’ accumulated more Zn in their roots and shoots by 31.4% and 19.9% than ‘Eleonora’, respectively. Furthermore, our results demonstrated that by biofortifying the nutrient solution with Zn it is possible to further increase its concentration in the edible part of basil cultivars. Indeed, supplementing the nutrient solution with Zn increased Zn shoot accumulation of ‘Aroma 2’ by 23.1% and ‘Eleonora’ by 9.7%, as overall average of Zn treatments, compared to the control. Similar studies have reported analogous results with other plant species (*Lactuca sativa* L., *Brassica oleracea* L. var. capitata, *Brassica oleracea* L. var. italica, and *Beta vulgaris* L.) when were exposed to Zn biofortified solutions (Sagardoy et al., 2009; Padash et al., 2016; White et al., 2018). However, in our study the increase in Zn concentration in basil tissues (root, shoot) was not proportional to the Zn concentration increase in the nutrient solution. In addition, Zn bioaccumulation in the roots of both cultivars was on average 26% higher than in the shoots (overall average of Zn treatments and the Control). This behavior has been cited for other Zn non-hyperaccumulative plants such as *Oryza sativa* and *Beta vulgaris* L. (Sagardoy et al., 2009; White and Broadley, 2011). It seems that this behavior is regulated by non-hyperaccumulative plant genes such as the ZIP, HMA, MTP, ZIF1, and FRD3. These genes, which are involved in Zn concentration, sequestration and redistribution in the plant, are up-regulated only under Zn deficiency, a condition that was not observed in this trial (White and Broadley, 2009). Conversely, concentration, translocation and tissue bioaccumulation of Zn are maximized when hyperaccumulator species are exposed to Zn surplus conditions due to the substantially higher expression of the abovementioned genes (White and Broadley, 2011). Concerning basil, our results showed that basil Zn biofortification potential is also highly influenced by cultivar. Specifically, in this study, supplementing the nutrient solution with additional Zn had a much greater effect on ‘Aroma 2’ plant shoot Zn levels, which were approximately 138.8% more than those of ‘Eleonora’, compared to the control. Given this and the fact that after the application of the maximum Zn level (50 MM), accumulation in the shoots of ‘Eleonora’ was lower even than the shoots of ‘Aroma 2’ control plants, underlines the crucial role of cultivar selection in the expected results for biofortification programs of non-hyperaccumulative species. This conclusion is in agreement with other studies concerning basil biofortification trials with selenium and three lettuce genotypes biofortified with Zn in soil trials (De Almeida et al., 2020; Szerement et al., 2021). It is worth mentioning that Zn concentrations achieved in the two basil cultivars used in this study were similar to those obtained from three lettuce cultivars biofortified in soil cultivation with 30 mg kg^–1^ of ZnSO_4_ (De Almeida et al., 2020). This aspect highlights how, compared to soil cultivation, closed hydroponic systems offer a more rational management of key macro-micronutrients, while at the same time being able to achieve the intended Zn concentrations (15-30 mg kg^–1^ dw) (White and Broadley, 2011; Sahin, 2021).

Although both cultivars belong to the Genovese basil type, under control conditions ‘Aroma 2’ plants were more robust than ‘Eleonora’ plants, growing taller, with a greater number of leaves, higher plant leaf area, fresh biomass, total dry biomass, and dry matter than ‘Eleonora’ plants. It is worth noting that, under control conditions, the observed cultivar differentiation in terms of morphological characteristics (fresh biomass, number of leaves, and leaf area) could have a decisive effect on the Zn concentration and accumulation capacity of the two basil cultivars (Waters and Grusak, 2008; Impa et al., 2013; Gupta et al., 2016; Mengist et al., 2021). The latter becomes particularly important since it is reported that Zn distribution across Zn sinks could be regulated by plant morphological characteristics and Zn accumulation could be decreased under conditions that biomass is reduced (Gupta et al., 2016). Nevertheless, both basil varieties appeared to be susceptible when were exposed to the exceeding levels of Zn. Basil response to additional Zn was manifested by the alteration of morphological traits (plant height, leaf number, leaf area, fresh biomass), leaf CIELAB color, physiological traits (total chlorophyll, ACO_2_, gs, E, SPAD index, and Fv/Fm) and antioxidant activity (DPPH, FRAP, ABTS). The previous response of basil plants after Zn application denotes a typical reaction of plants under heavy metal stress (Tsonev and Cebola Lidon, 2012). Typical visual symptoms of altered plant growth due to Zn toxicity are growth inhibition, chlorosis of young leaves and cell death probably as a consequence of inhibition of DNA synthesis and inevitably cell mitosis (Sturikova et al., 2018). As it turns out, the Zn stress conditions plants were subjected to in this study were mild since no such severe visual symptoms were observed in any of the two cultivars. Furthermore, under these mild stress conditions, depending on the Zn level, a decrease in fresh biomass from 3.80-9.72% for ‘Aroma 2’ to 8.9-29.9% for ‘Eleonora’ could be observed, although the plants of both cultivars remained marketable without particular problems in their appearance. What was observed visually was that Zn surplus made ‘Eleonora’ plants appear more greenish compared to the control (−a*, +h°) while Zn treatments (above 25 MM) lessened the greenness of ‘Aroma 2’ plants (+a*, −h°). However, it is noted that the ‘Aroma 2’ cultivar was more tolerant to Zn surplus since plant leaf area, leaf number, and fresh biomass decreased on average by 32.3%, 38.6%, and 57.18%, respectively, less than the plants of ‘Eleonora’ compared to the control and overall Zn treatments. Most likely, as in the control solution, the higher number of leaves and fresh biomass of ‘Aroma 2’, acted as Zn sinks and could also account for the higher Zn accumulation in this cultivar compared to ‘Eleonora’ under Zn excess (Sturikova et al., 2018).

In our study, the observed variation in yield attributes of basil under Zn stress is probably related to the efficiency of the photosynthetic mechanisms of the two cultivars. Photosynthetic efficiency of plants is reduced under abiotic stresses, such as heavy metal stress, due to their negative effect on photosystems performance, electron transport mechanisms, gas exchange parameters, chlorophyll, and other photosynthetic pigments biosynthesis (Paunov et al., 2018; Sharma et al., 2020a). Specifically, Zn can inactivate chlorophyll by replacing Mg from the porphyrin head of the chlorophyll molecule during photosynthesis (Sharma A. et al., 2020). Furthermore, Zn exposure has been found to disturb the energy migration from the antenna complexes to the chlorophyll of the PSII reaction centers and could be responsible for inactivation of a part of the reaction centers (Paunov et al., 2018). The above can explain to a certain degree the decline of total chlorophyll content, SPAD index, and Fv/Fm of both cultivars in our experiment under Zn stress. Noteworthily, in our study and under control conditions, photosynthesis related features of the two cultivars were similar for almost all the parameters examined (except for total chlorophyll content and ACO_2_). However, adding Zn to the nutrient solution stressed ‘Eleonora’ plants physiology to a greater extent than ‘Aroma 2’ plants when compared to the control. Total chlorophyll concentration, stomatal conductance, and SPAD index of ‘Eleonora’ were reduced to a greater extent than ‘Aroma 2’, by 44.2%, 23.8% and 63.9%, respectively overall Zn treatments. In addition, transpiration rate of ‘Eleonora’ was reduced on average by 19.9% (above 12.5 MM Zn) and Fv/Fm appeared to decrease with increasing Zn concentration while transpiration rate of ‘Aroma 2’ was not affected and Fv/Fm decreased only up to the 25MM Zn level and thereafter remained the same. It must be noted though, that Fv/Fm of both cultivars fell within the optimal range (~0.80) for basil (Aldarkazali et al., 2019).

A factor related to photosynthesis that could provide an explanation for the different responses of the two cultivars is the production of reactive oxygen species (ROS) under Zn surplus. Excessive ROS production under heavy metal stress conditions, such as Zn stress, has been associated with photosynthetic rate limitation and thus plant biomass reduction (Alia and Pardha Saradhi, 1995; Sharma et al., 2007; Jain et al., 2010). On the other hand, ROS play an important role as signaling molecules by regulating numerous biological processes including response to abiotic stresses (Del Río, 2015; Singh et al., 2016). In this study, cultivar tolerance to Zn surplus was probably related to their antioxidant system efficiency and therefore the ability of each cultivar to balance the cellular ROS level, maintaining the essential redox homeostasis thus preventing extreme oxidative stress conditions (Singh et al., 2016). Indeed, our results showed that the antioxidant activity of both cultivars increased at almost all Zn levels compared to the control. Likewise, studies on *Pisum sativum* L., *Brassica oleracea* L. var. botrytis, *Brassica oleracea* L. var. italica, *Solanum lycopersicum* L., *Brassica nigra* L., and *Phaseolus vulgaris* L. demonstrated increased plant antioxidant activity under Zn surplus regardless of the cultivar used (Singh et al., 2013; Lingyun et al., 2016; Zafar et al., 2016; García-Gómez et al., 2017). It was suggested that increased Zn bioaccumulation in plant tissues could act as an activator of antioxidant cofactor enzymes and thereby increase plant antioxidant capacity (Zhu et al., 2013; Messias et al., 2015). However, in this study the fact that the addition of Zn increased the antioxidant activity of ‘Eleonora’ much more than that of ‘Aroma 2’ (159.7%, 229%, and 188.8% for DPPH, FRAP, and ABTS, respectively) without any yield benefits for ‘Eleonora’ confirms its greater sensitivity and consequently lower adaptability to excess Zn.

To prevent ROS oxidative damage, plants have several antioxidant mechanisms with overlapping functions at their disposal (Foyer, 2018). The latter include highly efficient antioxidant carotenoid or phenolic compounds that could provide enormous flexibility in redox control (Foyer, 2018). Carotenoids, such as lutein and *β*-carotene, are effective chloroplast antioxidants located in close proximity to chlorophylls. At the same time, however, their bioactive oxidized products can induce changes in gene expression that lead to acclimatization to abiotic stress conditions (Havaux, 2014). In our study, carotenoid production by the two cultivars was not always similar under Zn treatments. When plants were exposed to the highest Zn treatments (37.5-50 MM) both lutein and *β*-carotene content of ‘Aroma 2’ increased while only *β*-carotene (at 25 and 50 MM) concentration of ‘Eleonora’ improved when compared to the control. The latter could relate to the increased SPAD index and therefore the photosynthetic efficiency of ‘Aroma 2’ compared to ‘Eleonora’, especially at the higher Zn concentrations. Polyphenolic compounds may have also played an important role in controlling ROS over-production under Zn stress in our study. Chlorophyll concentration reduction under Zn excess and especially the increased production of ROS could have signaled the production of phenolic compounds (Del Río, 2015) since both basil cultivars’ total phenolic concentration increased in comparison to the control. An important role of phenols is their action as scavengers of free radicals to protect the plant from oxidative stress (Granato et al., 2018). In our study total phenolic content demonstrated a positive correlation with DPPH (r= 0.84, p<0.001), FRAP (r= 0.94, p<0.001), and ABTS (r= 0.82, p<0.001) antioxidant activities, while it appeared to have a negative correlation with total chlorophyll concentration (r= −0.71, p<0.001). Similar studies on lettuce (Blasco et al., 2008; Smoleń et al., 2017) and basil (Incrocci et al., 2019; Kiferle et al., 2019) have reported an increase in total phenolics after biofortification with iodine in soilless systems. More support for our results is provided by the role of Zn as a cofactor of crucial amino acids in secondary metabolic pathways such as shikimate dehydrogenase (SKDH), phenylalanine ammonia lyase (PAL) and polyphenol oxidase (PPO). Recent studies on *Brassica oleracea* L. cv. Bronco and *Coriandrum sativum* L. with Zn integration have observed an increase in aromatic amino acids such as phenylalanine, tryptophan, and tyrosine, which are essential precursors of auxin and salicylate and enzyme regulators of the secondary metabolic pathway (Marichali et al., 2014; Barrameda-Medina et al., 2017).

It is remarkable that under the highest Zn biofortification treatment (50 MM) both carotenoid (lutein and *β*-carotene) and phenolic concentration of ‘Eleonora’ were enhanced and reached higher levels to those of ‘Aroma 2’ control. The latter is very crucial because it could enable us to bio-enhance commercial cultivars whose low concentration of certain compounds beneficial to human health is determined genetically. The health benefits of carotenoids for humans include their general role as antioxidants and the prevention of degenerative macular diseases (Rao and Rao, 2007). Polyphenols have also a well-known antioxidant activity and play an important role in human nutrition. Studies on basil widely support the impact of genotype on the biosynthesis and bioaccumulation of individual phenolic acids. In a recent study, Ciriello et al. (2021a) found a higher concentration of rosmarinic acid in basil cultivars ‘Aroma 2’ and ‘Italiano Classico’ and of chicoric acid in ‘Eleonora’. In a similar study the same authors reported comparable results in Genovese basil cultivars, showing a significant cultivar-dependent response to cichoric and rosmarinic acid genotype (Ciriello et al., 2021a). Kwee and Niemeyer (2011) reported lower concentrations of chicoric acid in *Ocimum basilicum* × *Ocimum americanum*, in contrast to what was observed in *Ocimum basilicum* var. thyrsiflorum. The investigation of phenolic profiles in our study confirmed the influence of genotype on the biosynthesis of phenolic compounds, revealing preferential bioaccumulation of chicoric acid regardless of the cultivar. Chicoric acid is crucial for plants’ protection against insects, viruses, bacteria, fungi, and nematodes, and for humans in exerting antitumor, anti-obesity, antiviral, antidiabetic, and inhibitory functions of HIV integrase by inhibiting its replication (Lee and Scagel, 2013).

## Conclusions

In this study, we evaluated the potential of producing Genovese basil biofortified with Zn by adopting an agronomic strategy of increasing Zn integration in the nutrient solution (12.5, 25, 37.5, and 50 MM). The management of Zn in the floating raft system increased the concentration of Zn in the ‘Aroma 2’ and ‘Eleonora’ basil cultivars while negatively impacting their yield and physiology. However, increasing Zn in the nutrient solution significantly increased antioxidant activity, carotenoid, and polyphenol concentrations. Specifically, the best compromise between yield, phytochemical quality and zinc accumulation in leaves was observed in Aroma 2 biofortified with the highest zinc doze (50 µM). The highest tissue zinc levels in Aroma 2 × 50 (160.12 mg g^–1^ dw) resulted in a yield reduction of less than 10%, but a 15% increase in total phenolics. However, the strong cultivar-dependent response observed in the present study suggests that in biofortification programs for non-hyperaccumulative crops, the varietal choice is crucial in order to maximize the accumulation of this essential micronutrient and minimize yield loss. However, the consumption of biofortified Genovese basil with Zn can increase the intake of Zn by consumers while providing a product enriched concomitantly in valuable phytochemicals such as carotenoids and polyphenols.

## Data availability statement

The raw data supporting the conclusions of this article will be made available by the authors, without undue reservation.

## Author contributions

MC and YR: conceptualization and project administration. MC, LF, MK, GS, and GG: methodology, validation, formal analysis, investigation, and writing—original draft preparation. MC and LF: software. SP and YR: resources. MC and LF: data curation. MC, LF, MK, GS, and YR: writing—review and editing. YR: visualization. YR: supervision. SP: funding acquisition. All authors contributed to the article and approved the submitted version.

## Funding

This research was conducted in the framework of a PhD project sponsored by the Italian Ministry of Education (PON research and innovation).

## Acknowledgments

The authors are grateful to Dr. Christophe El-Nakhel for his technical assistance during the experiment.

## Conflict of interest

The authors declare that the research was conducted in the absence of any commercial or financial relationships that could be construed as a potential conflict of interest.

## Publisher’s note

All claims expressed in this article are solely those of the authors and do not necessarily represent those of their affiliated organizations, or those of the publisher, the editors and the reviewers. Any product that may be evaluated in this article, or claim that may be made by its manufacturer, is not guaranteed or endorsed by the publisher.
